# Complementary feeding practices among mothers having children less than two years old attending well-baby clinics in Jazan City, Saudi Arabia

**DOI:** 10.11604/pamj.2023.45.45.35451

**Published:** 2023-05-17

**Authors:** Aisha Awaf, Anas Elias, Mohamed Salih Mahfouz

**Affiliations:** 1 Department of Family Medicine, Ministry of Health, Jazan, Saudi Arabia,; 2 Department of Community Medicine, Faculty of Medicine, Jazan University, Jazan, Saudi Arabia

**Keywords:** Infant, breastfeeding, feeding practices, knowledge

## Abstract

**Introduction:**

infant feeding practices are important determinants of growth and development not only in infancy but also in later life. The main objective of this study is to describe infant feeding practices and the factors affecting complementary feeding (CF) among mothers in Jazan City.

**Methods:**

an observational cross-sectional study was conducted among 400 mothers having children less than 24 months old, attending the well-baby clinics in Primary Health Care Centers (PHCCs) in Jazan City, Saudi Arabia. A structured questionnaire was used to collect data in a face-to-face interview.

**Results:**

on the mother’s level of knowledge regarding proper infant feeding, 49% scored high, 41% scored medium, and 9.75% scored low. The mother’s educational level, working status, family income, and knowledge source played a significant role in the knowledge scores (p < 0.05 for all). Overall, 15.8% of the mothers never breastfed (BF) their children, 39.8% BF less than six months, 17.5% BF 6-12 months, and 27% BF over 12 months. Breastfeeding feeding (BF) duration is significantly associated with maternal literacy and family income (p = 0.006 for both). Overall, approximately one-third of the women (36.2%) started CF at six months or later, while 63.8% started CF at 4-6 months.

**Conclusion:**

Jazan women use some positive infant feeding practices, including breastfeeding. One-third of the women started CF at six months or later, which is considered the optimal time for the CF introduction. More nutrition education is necessary to raise maternal awareness regarding appropriate infant feeding and weaning practices.

## Introduction

Adequate nutrition during infancy and early childhood is essential to ensure the growth, health, and development of children. Poor diet increases the risk of illness, and is responsible, directly or indirectly, for one-third of the estimated 9.5 million deaths of children under five years of age [1]. It is well known that the period from birth to two years of age is a “critical determinant” optimal growth, health, and behavioral development. WHO recommends exclusive breastfeeding from birth to 6 months of age and the introduction of complementary foods at six months of age while continuing to breastfeed [2]. The American Academy of Pediatrics (AAP) recommends exclusive breastfeeding for the first 4 to 6 months of age, and continued breastfeeding for the first year of life and beyond, if possible, with the introduction of solid foods at 4 to 6 months of age [3].

Complementary feeding practices have a detrimental impact on a child's growth, health, and development in the first two years of life. Inappropriate complementary feeding leads to child malnutrition, which contributes to stunting and underweight children [4]. The introduction of solid foods at six months of age remains a desirable goal for many countries, while it is advisable to introduce solid foods together with breast milk before that age [5]. It is not advisable to delay the introduction of potentially allergenic foods or gluten to prevent the development of allergic diseases. There is no ideal timing for gluten introduction in relation to the onset of coeliac disease (CD) and type 1 diabetes mellitus (T1DM) [6].

Early complementary feeding, between 5 and 6 months, has been regarded as a child nutrition problem in Latin America, the Caribbean, East Asia, and the Pacific, where almost half of the children consume solids at this age [7]. Studies on complementary feeding in the Middle East and North Africa (MENA) region are relatively scarce. The available evidence suggests that mixed breast- and bottle-feeding are predominant in the first months and that complementary foods are introduced prematurely. The early introduction of non-milk fluids has been described as a common practice in the region. The early introduction of complementary foods has been reported in as many as 80% of cases in several countries [8]. The situation in the Kingdom of Saudi Arabia (KSA) is similar. A nationwide cross-sectional survey suggests that in KSA, about 76.1% of mothers had introduced bottle-feeding by three months, and 48.3% cited insufficient milk as the reason for introducing the bottle [9]. Another survey indicated that solid foods were introduced to 81.5% of infants between 4 to 6 months of age [10].

While many studies have previously been conducted in all parts of Saudi Arabia [9,10], there is a lack of literature on complementary feeding practices in the Jazan region. The main objective of this study is to assess the knowledge, attitude, and practices related to complementary feeding and identify the factors that may affect this practice among mothers of children under 24 months in PHCCs in Jazan City. Answering questions regarding knowledge, practices, and their associated factors among mothers will help policymakers develop effective strategies and policies concerning complementary feeding practices of children under two years.

## Methods

**Study design, area, and population:** we conducted an observational cross-sectional study among mothers attending the well-fare baby clinics in Gizan PHCCs between February and April 2018. The research was conducted in Gizan City, the capital of the Jazan province, which is located in the southwestern part of the Saudi Arabian Peninsula. According to the 2010 census, the region´s population is over 1.5 million. The study targeted mothers attending well-baby clinics in PHCCs for growth monitoring and immunization. Inclusion criteria involved women with a full-term child under 24 months. Women who did not consent to the study and those who could not communicate in Arabic were excluded.

**Sampling procedures:** the sample size for this research was calculated building on the statistical formula:


$$n=\frac{Z^{2}*p*q}{d^{2}}$$

The parameters of the formula include n: the initial sample size; p: is an anticipated population proportion. Z: is the standardized variable that corresponds to a 95% confidence level; d: is the desired marginal error. Since no estimate for the level of knowledge about complementary feeding among women, it is safer to set the prevalence at 50%. Based on the values P = 0.5, d the desired marginal error = 0.05, and z = 1.96. The initial sample will be 383 women. The initial sample was further increased by 10 % to account for the non-response, providing a final sample of 422 mothers. Using simple random sampling, five PHCCs were selected out of 18 PHCCs. The total sample size was distributed equally to the five selected PHCCs. In the final stage, women were recruited using systematic random from each selected PHCCs.

**Methods of data collection and study tool:** data were collected through personal interviews with a predesigned structured questionnaire designed after consulting relevant literature [7-11]. An Arabic-speaking trained nurse conducted the interviews and collected the data. The final version of the questionnaire consisted of 42 closed-ended and open-ended short-answer questions. The questionnaire questions were categorized into three sections: (1) socioeconomic and demographic variables; (2) Maternal knowledge about infant feeding and sources of information and (3) infant feeding practices related information and women's attitudes toward infant feeding. A pilot study was carried out among 20 participating mothers to test the questionnaire's reliability. Adjustments were made after feedback was gathered and evaluated. The pilot study established that the questionnaire took an average of 15 minutes to complete and had a reasonable Cronbach's alpha. The participants from the pilot study were not included in the final study sample.

**Operational definitions and study variables:** complementary feeding was defined as introducing solid, semi-solid, or soft foods during the first six months of age. Exclusive breastfeeding was defined as the proportion of infants 0-5 months of age who were fed no other food or drink, not even water, except breast milk (including milk expressed or from a wet nurse), but allowed the infant to receive oral rehydration salt, drops, and syrups (vitamins, minerals, and medicines). The mother´s level of knowledge regarding infant feeding was assessed using knowledge items in the questionnaire (10 questions). One score was given to each correct response, while incorrect responses were scored 0. A total knowledge score was computed by summation of individual responses for each participant. High knowledge was defined as a total score equal to or above 75%. Women with a score of less than 50% indicate a low level of knowledge. Independent variables involve first socio-demographic factors like maternal factors, including age, education and occupation, number of children and family monthly income-second child-related factors involving child age, gender and weight. Dependent variables were appropriate complementary feeding practices, mothers´ level of knowledge regarding infant feeding, exclusive breastfeeding, and breastfeeding duration.

**Data analysis:** the computer program IBM SPSS Statistics for Windows, version 24.0 program (IBM Corp., Armonk, N.Y., USA) was used to enter and analyze data. Socio-economic and demographic, maternal and child characteristics data were tabulated, and the percentages and frequencies were calculated. A chi-squared test assessed the association between the maternal and child factors and the dependent variables. Logistic regression was used to determine the factors associated with complementary feeding among mothers. The odds ratio and their 95% CIs were calculated. A p-value less than 0.05 was used to indicate statistical significance.

**Ethics approval and consent to participate:** this study was conducted according to the ethical standards of Saudi Arabia. All the participants read, understood, and signed the written consent form prepared by the study team. The Jazan Health Directorate ethical committee approved the study protocol. Participants were told at the beginning that they could choose to participate or withdraw from the study at any time.

## Results

The response rate for this survey was 94.8% (400 out of 422). The majority of mothers, 56.5% (n = 226), were 26-35 years old, while 25.8% (n = 103) were 18-25 years old. Almost 61% (n = 244) had a university-level education, and 25.5% (n = 102) had a secondary-level education. Among the mothers, 68.5% (n = 274) did not work, while 31.5% (n = 126) were working. Among the 400 infants, 51% (n = 204) were males and 49% (n = 196) were females. Mothers´ primary sources of information varied; about one-third of the mothers, 31.8% (n = 127), received their knowledge about infant feeding from the family; 21.3% (n = 85) from social media; 14.2% (n = 57) from the health sector; 18.3% from mixed sources; and 14.5% (n = 58) did not receive any information about infant feeding (Table 1).

**Table 1:** background characteristic of the mothers and their children (n=400)

Variables		
**Child’s characteristics**		
**Child’s gender**	**N**	**%**
Male	204	51.0
Female	196	49.0
**Child’s age**		
Less than 1 month	5	1.3
1-6 months	91	22.8
7-12 months	101	25.3
12-24 months	203	50.7
**Child’s weight**		
Less than 6 kg	67	16.8
6-11 kg	267	66.8
More than 11 kg	66	16.5
**Mother’s characteristics**		
**Mother’s age (years)**		
15-25	106	26.5
26-35	226	56.5
36 and above	68	17.0
**Level of education**		
Illiterate	4	1.0
Primary	16	4.0
Intermediate	34	8.5
Secondary	102	25.5
University	244	61.0
**Occupation**		
Working	126	31.5
Non-working	274	68.5
**Family characteristics**		
**Family income**		
Less than 5,000 SR#*	76	19.0
5,000-10,000 SR	185	46.3
More than 10,000 SR	139	34.8
**Number of children**		
1 child	119	29.8
2-3 children	179	44.8
4 children and more	102	25.5

#SR = Saudi Riyal equivalent to 0.27 $

As shown in Table 2, the mothers´ level of knowledge regarding infant feeding was distributed in the following way: low 9.75%, medium 41%, and high 49%. The mother´s level of knowledge by age showed no statistically significant difference (p = 0.617). However, the mother´s education played a significant role (p = 0.000), where 59.8% (n = 146) of women with university-level education had the highest knowledge score, whereas 0% (n = 0) of women who were illiterate or had a primary school level education got a high score. Working mothers had a slightly higher percentage of high scores compared to non-working mothers: 50.0% vs. 48.7%, respectively. Family income also gave a slight advantage, where 52.5% of mothers with a monthly income level of over 10,000 Saudi riyals had a high level of knowledge. From the various sources of information, social media had the largest impact, where 60% of the mothers who used social media scored high, and only 3.5% scored low. Surprisingly, women who received information from the health sector scored worse, with only 35.1% of them scoring a high level of knowledge and 10.5% scoring low.

**Table 2:** mother’s level of knowledge regarding infant feeding (n=400)

Maternal variables	Knowledge score			P-value
	Low	Medium	High	
**Mother’s age (years)**				
15-25	12 (11.4)	46 (43.8%)	47 (44.8%)	0.207
26-35	18 (8.0%)	86 (38.1%)	122 (54.0%)	
36 and above	9 (13.2%)	32 (47.1%)	27 (39.7%)	
**Mother’s education**				
Illiterate	4 (100.0%)	0 (0.0%)	0 (0.0%)	<0.001
Primary	9 (56.2%)	7 (43.8%)	0 (0.0%)	
Intermediate	17 (50.0%)	15 (44.1%)	2 (5.9%)	
Secondary	9 (8.9%)	44 (43.6%)	48 (47.5%)	
University	0 (0.0%)	98 (40.2%)	146 (59.8%)	
**Mother’s occupation**				
Working	2 (1.6%)	61 (48.4%)	63 (50.0%)	0.001
Non-working	37 (13.6%)	103 (37.7%)	133 (48.7%)	
**Family income**				
Less-5,000 SR#	17 (22.7%)	26 (34.7%)	32 (42.7%)	0.001
5,000-10,000 SR	16 (8.6%)	78 (42.2%)	91 (49.2%)	
More-10,000 SR	6 (4.3%)	60 (43.2%)	73 (52.5%)	
**Source of information**				
Family	17 (13.5%)	40 (31.7%)	69 (54.8%)	0.010
Social media	3 (3.5%)	31 (36.5%)	51 (60.0%)	
Health sector	6 (10.5%)	31 (54.4%)	20 (35.1%)	
Did not receive any information	8 (13.8%)	27 (46.6%)	23 (39.7%)	
From more than one source	5 (6.8%)	35 (47.9%)	33 (45.2%)	
**Overall prevalence**	39 (9.75%)	164 (41%)	196 (49%)	

P-value based on Chi-square test; #SR = Saudi Riyal equivalent to 0.27 $

Table 3 presents the overall understanding of women regarding infant feeding practices. Only 11.8% (n = 47) of the mothers strongly believe that it is not necessary to feed the baby the milk from the first two days, while 51.7% (n = 207) believe in the importance of the colostrum. Almost 65.5% (n = 262) of women believe that food other than milk must not be introduced before 4 months of age. The majority of the women 61.3% (n = 245) agree that breastfeeding is beneficial to both the mother and the baby.

**Table 3:** mother’s attitude toward some feeding practices (n=400)

Statement	Strongly disagree	Disagree	Neutral	Agree	Strongly agree
In the first 2 days after delivery, there is no need to breastfeed because there is little milk in the breast	207 (51.7%)	99 (24.8%)	8 (2.0%)	39 (9.8%)	47 (11.8%)
It is advisable to introduce foods other than breast milk before 4 months, as breast milk alone is not enough	262 (65.5%)	57 (14.2%)	16 (4.0%)	35 (8.8%)	30 (7.5%)
The mother benefits from breastfeeding as well as the child	11 (2.8%)	22 (5.5%)	30 (7.5%)	92 (23.0%)	245 (61.3%)
At the time of weaning, it is better to give different types of foods from the beginning, so that the baby would accept the different tastes	214 (53.5%)	99 (24.8%)	28 (7.0%)	46 (11.5%)	13 (3.3%)
If the child develops diarrhea, there is no need to change his or her food immediately	256 (64.0%)	103 (25.8%)	21 (5.3%)	18 (4.5%)	2 (0.5%)

When data were analyzed with respect to the feeding pattern (exclusive BF, formula feeding, or both) in the first 6 months, maternal variables such as literacy, occupation, age, and family income had no significant impact (p > 0.05, for all), as the pattern of child feeding during the first 6 months did not differ according to the different mentioned variables. However, overall, 18.3% (n = 73) were using exclusively BF, 26.3% (n = 105) were feeding formula, and 55.5% (n = 222) were using both (Table 4).

**Table 4:** patterns of child feeding during the first 6 months (n=400)

Maternal variables		Practices N (%)			P-value
		Exclusive breast-feeding	Formula feeding	Both (breast- and formula feeding)	
**Level of mother’s education**	Illiterate	1 (25.0%)	1 (25.0%)	2 (50.0%)	0.689
	Primary	3 (18.8%)	5 (31.2%)	8 (50.0%)	
	Intermediate	6 (17.6%)	14 (41.2%)	14 (41.2%)	
	Secondary	21 (20.6%)	24( 23.5%)	57 (55.9%)	
	University	42 (17.2%)	61 (25.0%)	141 (57.8%)	
**Mother’s age (years)**	15-25	20(18.9%)	34(32.1%)	52(49.1%)	0.521
	26-35	41 (18.1%)	53 (23.5%)	132 (58.4%)	
	36 and above	12 (17.6%)	18 (26.5%)	38 (55.9%)	
**Mother’s occupation**	Working	21 (16.7%)	31 (24.6%)	74 (58.7%)	0.674
	Non-working	52 (19.0%)	74 (27.0%)	148 (54.0%)	
**Family income**	Less than 5,000 SR#	18 (23.7%)	23 (24.9%)	35 (46.1%)	0.451
	5,000-10,000 SR	31 (16.8%)	46 (20.4%)	108 (58.4%)	
	More than 10,000 SR	24 (17.3%)	36 (25.9%)	79 (56.8%)	
**Overall**		73 (18.3%)	105 (26.3%)	222 (55.5%)	

P-value based on Chi-square test; #SR = Saudi Riyal equivalent to 0.27 $

Table 5 shows that the duration of BF is significantly associated with maternal education (p = 0.006), as 75.0% of illiterate women breastfed their children for less than 6 months compared to 39.3% of women with university education. Overall, 15.8% of children are never BF; 39.8% are BF for less than 6 months; 17.5% are BF for 6-12 months; and 27% are BF for over 12 months. Table 6 presents the association between complementary feeding practices and some selected variables. Overall, approximately one-third of the women (36.2%) started CF at 6 months or later, while 63.8% started CF at 4-6 months.

**Table 5:** breastfeeding duration among the mothers who participated in the survey (n=400)

Maternal variables		Less than 6 months	6 - 12 months	More than 12 months	Never breastfed	P-value
**Level of mother’s education**	Illiterate	3 (75.0%)	1 (25.0%)	0 (0.0%)	0 (0.0%)	0.006
	Primary	4 (25.0%)	0 (0.0%)	9 (56.2%)	3 (18.8%)	
	Intermediate	12 (35.3%)	3 (8.8%)	6 (17.6%)	13 (38.2%)	
	Secondary	44 (43.1%)	18 (17.6%)	26 (25.5%)	14 (13.7%)	
	University	96 (39.3%)	48 (19.7%)	67 (27.5%)	33 (13.5%)	
**Mother’s age (years)**	15-25	46 (43.4%)	20 (18.9%)	18 (17.0%)	22 (20.8%)	0.091
	26-35	91 (40.3%)	40 (17.7%)	65 (28.8%)	30 (13.3%)	
	36 and above	22 (32.4%)	10 (14.7%)	25 (36.8%)	11 (16.2%)	
**Mother’s occupation**	Working	53 (42.1%)	24 (19.0%)	34(27.0%)	15 (11.9%)	0.527
	Non-working	106 (38.7%)	46 (16.8%)	74 (27.0%)	48 (17.5%)	
**Family income**	Less than 5,000 SR	22 (28.9%)	15 (19.7%)	24 (31.6%)	15 (19.7%)	0.006
	5,000-10,000 SR	85 (45.9%)	33 (17.8%)	40 (21.6%)	27 (14.6%)	
	More than 10,000 SR	52 (37.4%)	22 (15.8%)	44 (31.7%)	21(15.1%)	
**Overall duration**		159 (39.8%)	70 (17.5%)	108 (27.0%)	63 (15.8%)	

P-value based on Chi-square test; #SR = Saudi Riyal equivalent to 0.27 $

**Table 6:** factors associated with complementary feeding among mothers who had 0- to 23-month-old children in Jazan

Maternal variables	Complementary feeding practice		COR (95% CI)
	Appropriate N%	Inappropriate N%	
**Knowledge score**			
Low	12 (30.8)	27 (69.2)	1
High	133 (36.8)	228 (63.2)	1.3 (0.6-2.7)
**Occupation**			
Working	51 (40.5)	75 (59.5)	1.3 (0.8-2.0)
Non-working	94 (34.3)	180 (65.7)	1
**Income level**			
Less than 5,000 SR	23 (30.3)	53 (69.7)	1
5,000-10,000 SR	68 (36.8)	117 (63.2)	1.5 (0.8-2.6)
More than 10,000 SR	54 (38.8)	85 (61.2)	1.1 (0.7-1.7)
**Child’s gender**			
Male	75 (36.8)	129 (63.2)	1.04 (0.7-1.6)
Female	70 (35.7)	126 (64.3)	1
**Child’s weight**			
Less than 6 kg	22 (32.8)	45 (67.2)	1
6-11 kg	101 (37.8)	166 (62.2)	1.02 (0.5-2.1)
More than 11 kg	22 (33.3)	44 (66.7)	0.82 (0.5-1.5)
**Overall**	145 (36.2)	225 (63.8)	

# COR: crude odds ratio; CI: confidence interval; Kg =kilogram

**Figure 1:**
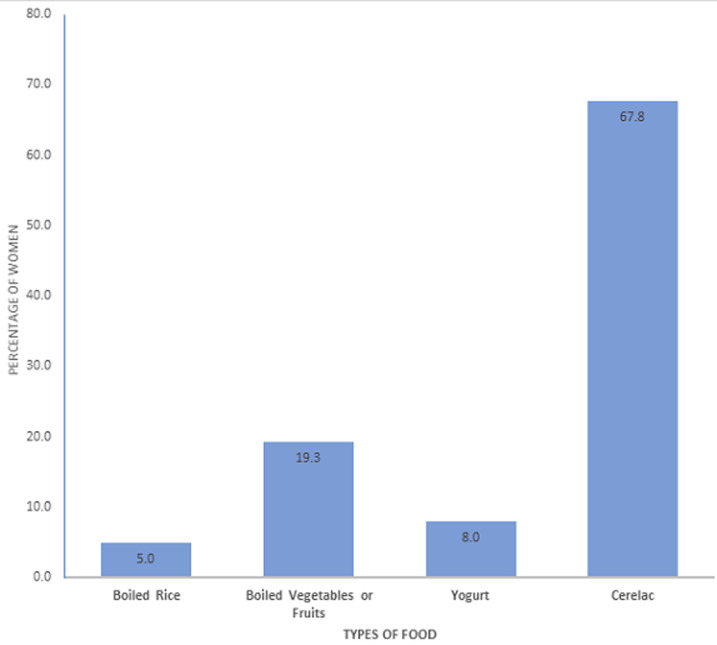
introduction of complementary feeding for the first time (actual or planned)

**Figure 2:**
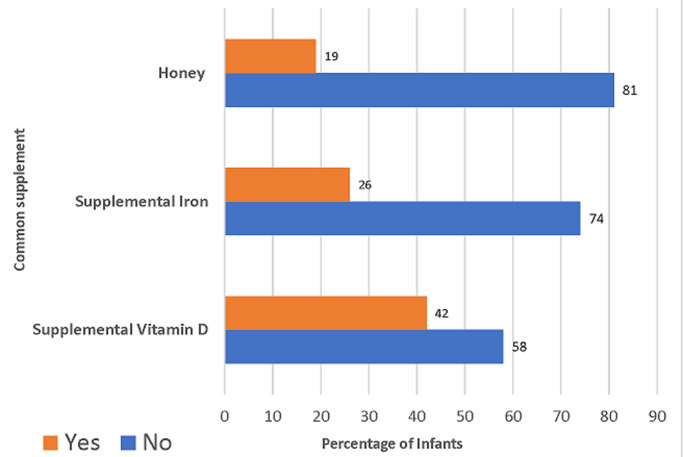
complementary feeding during the first year among the infants

Figure 1 demonstrates the introduction of complementary feeding. The foods commonly introduced were Cerelac (67.8%), boiled vegetables or fruits (19.3%), yogurt (8%), and boiled rice (5%). Figure 2 shows that vitamin D is the most common supplement given to infants under 1 year of age, used by 42% of mothers (n = 168), followed by iron 19% (n = 76). Around a quarter of mothers (26%) introduced honey to their infants before 1 year of age (n = 104), which is against the recommendations.

## Discussion

Breastfeeding is the best method of feeding an infant, with short- and long-term benefits for the mothers, the babies, the economy, and the environment [12]. The World Health Organization (WHO) recommends exclusive BF until six months of age [1]. It is further recommended that BF should continue until two years of age, along with other nutritionally adequate complementary foods. This study tries to evaluate the knowledge, attitudes, and practices related to complementary feeding, and to identify the factors that might affect it, among mothers who attended the immunization clinics or well-baby clinics in PHCCs in Jazan City.

The results reveal that the mother´s level of education plays a significant role, where women with a university-level education had the highest knowledge score, whereas the 0% of women who were illiterate or had a primary school level education got a high score. It is well documented that the mother´s education level is an essential determinant of infant feeding practices [13-16]. An Egyptian study on breastfeeding and weaning documented that the mother´s education was significantly associated with feeding, but that there was no association of feeding with the mother´s age at the child´s birth, occupation, or place of birth [17]. Working mothers had a slightly higher percentage of high knowledge scores than non-working mothers (50.0% vs. 48.7%, respectively). The family income level also gave a slight advantage, where 52.5% of women with monthly income levels of over 10,000 Saudi riyals scored high, while 22.7% of women with a monthly income below 5,000 Saudi riyals scored low. These findings are consistent with much of the literature [15,17].

Overall, 15.8% of children are never BF, 39.8% are BF for less than six months, 17.5% are BF for 6-12 months, and 27% are BF for over 12 months. These results are slightly different from a study on breastfeeding indicators conducted in Jazan in 2012, where only 7% of women were never breastfed [18]. Almost 65.5% (n = 262) of women believe that food other than milk must not be introduced before four months of age, and 64% (n = 256) agree with changing the baby´s diet if diarrhea develops (which is consistent with a study conducted in Riyadh [19]). Overall, the majority of the women (63.8%) started CF at 4-6 months, and 36.2% started after six months. This pattern is different from another study conducted in KSA, which found that 81.5% of KSA infants received CF at 4-6 months of age [10], and consistent with a cross-sectional study conducted in Tabuk in 2015, which found that 62.5% of infants are given solid foods prior to 4 months of age [20]. In other Middle Eastern countries, there is a large variation between the countries regarding the introduction of CF [7]. The percentage of infants who receive complementary foods before 6 months is 78.6% in Iraq, 70% in the Emirates, 52.9% in Lebanon, 35% in Turkey, and 30.4% in Kuwait [21-25].

In our study, around a quarter of mothers (26%) introduced honey to their infants before the first year, which is against the recommendations. Evidence shows that honey is one of the main sources of botulism, especially infant botulism. Most infant botulism patients are between 6 weeks and 6 months of age [26]. Vitamin D is the most common supplement given to infants under one year of age. The American Academy of Pediatrics (AAP) recommends 400 IU of oral vitamin D supplement per day for infants who are either breastfed or consuming less than 1 liter of infant formula per day [27]. Unfortunately, only 19% (n = 76) of the infants in our study were supplied with iron. There is increasing evidence of iron deficiency anemia in infants, especially infants on BF and those not on the milk-fortified formula [28]. A cross-sectional study from the northwestern region of Saudi Arabia showed that 51% of infants aged 6-24 months were diagnosed with IDA [29].

In our study, many mothers indicated incomplete adherence to WHO recommendations for breastfeeding and infant feeding practices. Those conducting the intervention and further research should pay attention to factors such as cultural practices, access to and utilization of healthcare facilities, child-feeding education, and family planning. Surprisingly, women who received information from the health sector scored poorly, with only 35.1% in high and 10.5% in low, which is lower than what women who received no information scored (39.7% high). It is crucial to encourage more health education sessions for mothers antenatally and during each visit to well-baby clinics in the PHCCs.

Our study has some limitations, including the following: (a) the study was conducted in Jazan City only, and the results may not be generalizable to the entire Jazan region population; (b) as with many cross-sectional studies, the conclusions on causality are less definitive than with other types of studies; and (c) the women´s responses may be affected by recall bias. Despite these limitations, our research still provides a picture of the complementary feeding practices in Jazan and can be used in further intervention programs.

## Conclusion

Jazan women have some beneficial infant feeding practices, including breastfeeding. One-third of the women started CF at six months or later, which is considered the optimal time for CF introduction. The foods commonly introduced to infants included rice, vegetables, fruit, yogurt, and cereal. More efforts are needed in nutrition education to raise awareness of appropriate infant feeding and weaning practices.

### What is known about this topic

- *Complementary feeding practices have a detrimental impact on a child's growth, health, and development in the first two years of life;*
- *Inappropriate complementary feeding leads to child malnutrition, which contributes to the stunting and underweight children*.

### What this study adds

- *Women use some positive infant feeding practices, including breastfeeding;*
- *One-third of the women started CF at six months or later, which is considered the optimal time for the CF introduction;*
- *There is a need for nutrition education to raise maternal awareness regarding appropriate infant feeding and weaning practices*.
